# Exercise-Induced Changes in Tumor Growth via Tumor Immunity

**DOI:** 10.3390/sports9040046

**Published:** 2021-03-30

**Authors:** Polyxeni Spiliopoulou, Maria Gavriatopoulou, Efstathios Kastritis, Meletios Athanasios Dimopoulos, Gerasimos Terzis

**Affiliations:** 1 Sports Performance Laboratory, School of Physical Education and Sport Science, National and Kapodistrian University of Athens, 17237 Athens, Greece; gterzis@phed.uoa.gr; 2 Department of Clinical Therapeutics, School of Medicine, Alexandra General Hospital, National and Kapodistrian University of Athens, 11528 Athens, Greece; mariagabria@gmail.com (M.G.); ekastritis@gmail.com (E.K.); mdimop@med.uoa.gr (M.A.D.)

**Keywords:** cancer, physical activity, cancer immunity, leukocytosis, tumor infiltration

## Abstract

Immunity in the tumor microenvironment plays a central role in tumor development. Cytotoxic immune cells act against tumors, while tumors manage to trigger immunosuppressive mechanisms for defense. One bout of physical exercise acutely regulates the immune system inducing short-term redistribution of immune cells among body organs. Repeated acute immune cell mobilization with continuing exercise training results in long-term adaptations. These long-term exercise-induced changes in the immune system arise both in healthy and in diseased populations, including cancer patients. Recent preclinical studies indicate that physical exercise may have a positive impact on intra-tumoral immune cell processes, resulting in tumor suppression. This short narrative review describes the effect of physical exercise on tumor growth as detected via changes in tumor immunity. Research evidence shows that exercise may improve tumor-suppressive functions and may reduce tumor-progressive responses and mechanisms of immune cells, controlling tumor development. Specifically, it seems that exercise in rodents triggers shifts in tumor infiltration of macrophages, neutrophils, natural killer cells, cytotoxic and regulatory T lymphocytes, resulting in tumor suppression. These recent promising data suggest that physical exercise could be combined with anticancer immunotherapies, although exercise parameters like intensity, duration, and frequency need to be evaluated in more detail. More research is needed to investigate the effect of exercise in other immune cell subtypes and their possible connection with tumor growth, whilst information from human tumors is also required.

## 1. Introduction

Acute exercise, i.e., a single physical exercise bout, induces transient physiological changes resulting in tumor microenvironment modifications and possible alterations in tumor development. Exercise training, i.e., the long-term application of single physical exercise bouts, may interfere with tumor growth via four main physiological pathways: (i) tumor vascularization, (ii) cancer cell metabolism, (iii) myokine production, and (iv) immune function [1]. This review depicts the current understanding of long-term exercise effect on tumor development via alterations of the immune function. The importance of tumor immunity in tumor development has been long appreciated, with current data showing that more immunogenic tumors have better response to therapies [2]. Along this line, therapies targeting the immune response (immunotherapies) have been approved for several malignances. Acute exercise stimulates temporary immune responses. For example, during an exercise session on a treadmill at 80% of VO_2max_ until exhaustion, acute redistribution of lymphocytes among organs is observed [3,4], which is important for immune readiness [4]. Here, we summarize the current knowledge of exercise-mediated changes in tumor growth via changes in immune cell subtypes. The present descriptive review includes only studies which measured tumor growth during exercise and report on the possible changes related to immune cell functions. Studies reporting the effect of exercise on tumor immunity but not on tumor burden were not included. Also, changes in tumor growth explained by other exercise-induced changes, such as vascularization or cell metabolism, are not discussed. Mainly murine experimental models have examined this topic, while exercising programs included exclusively aerobic exercise such as wheel running, treadmill running, or swimming. Training parameters varied among studies. Training duration ranged between 2 and 12 weeks, and between 30 min per session and exercise until fatigue. Training frequency was 5–6 days per week or voluntary exercise frequency, while exercise intensity varied among studies.

## 2. Immune Response

The immune response is a composite process involving several cellular processes [5,6]. In cancer, the immune system is partly triggered by abnormal protein production expressed by the mutated DNA of tumor cells [7]. Phagocytosis of cancer cells via macrophages mobilizes immune cells such as neutrophils, which subsequently produce cytokines (mostly IL-6, INF-γ, TNF-α), activating natural killer (NK) and dendritic cells. NK cells can destroy cancer cells, while CD4^+^ cells contribute to anti-antigen surface receptor production by cytotoxic CD8^+^ cells. Similar to NK cells, cytotoxic CD8^+^ can destroy cancer cells [6]. Downregulation of the immune system has a vital role in healthy individuals primarily to prevent autoimmune diseases and allergies, although it is a negative prognostic marker for cancer. Downregulation includes immunosuppressive mechanisms such as the actions of regulatory T cells (Tregs) [6].

Immune cell subtypes have different phenotypes. Macrophages (M) and neutrophils (N) can be categorized as anti-antigen attack contribution cells (designated with the number 1, e.g., M1) or tissue-repairing cells (designated with the number 2, e.g., M2). After invasion, phenotype 1 immune cells focus on eliminating pathogens, while phenotype 2 cells repair damaged tissues. In cancer, tissue repairing corresponds to tumor development, and therefore, immune cells of the tumor microenvironment are labelled as “tumor suppression” cells (CD8^+^, NK, M1, N1, Th1, DC1) and “tumor progression” cells (M2, N2, Th2, DC2, Treg) [6].

## 3. Effect of Exercise on Tumor Growth as Explained by Changes in Tumor Immunity

### 3.1. The Role of Macrophages

Infiltration of macrophages in the tumor microenvironment is considered an adverse event for tumor growth: macrophages secrete epidermal growth factor receptor (EGFR) family ligands which finally trigger the production of signal transducer and activator of transcription 3 (STAT3) and NF-kB, both of which contribute to tumor development and resistance to therapy [8]. However, directed polarization of macrophages to the M1 phenotype induced reduction of tumor burden in mammals [9], while high tumor infiltration of M2 is correlated with worse clinical outcome [8], explaining why tumors reinforce polarization of macrophages to the M2 rather than to the M1 phenotype [5,10]. Previous data showed that physical exercise controls macrophages’ recruitment in mice with lack of peroxisome proliferator-activated receptors γ (PPARγ), a regulatory receptor for macrophages [11], which implies that exercise may be beneficial for macrophage antitumor response.

In an early study, two weeks of daily treadmill running 3 h/day or until fatigue with gradually increasing velocity (20 to 40 m/min at 5% grade) (Table 1) in rodents with allogenic EL-4 tumors (lymphoid cells), delayed tumor growth after two weeks, yet tumor volume was similar between the exercise and the control group [12]. Significant changes in tumor immunity were found on the 10th day of exercise, with the total number of macrophages being significantly lower in the tumors of the exercise group compared to the control group (Table 2) [12]. In another study, six weeks of swimming 5 days/week for 1 h/day at 50% of maximal workload (Table 1) in rodents injected with Ehrlich tumor cells reduced tumor growth, which was accompanied by reduced tumor infiltration of macrophages (Table 2) [13]. It was recently reported that two weeks of daily voluntary wheel running about 6 km/day, followed by injection of I3TC cells and eight more weeks of exercise (Table 1) resulted in diminished tumor growth, with no alterations in tumor infiltration of macrophages [14]. Twelve weeks of treadmill running 6 days/week, 1 h/day, at 15 m/min with −5% grade (Table 1) in mice with colon cancer induced reduction in tumor burden, reduction in the expression of total macrophages, and reduction in three of the M2 markers (CD206, IL-10, IL-4, CCL17, CCL22), while the effect of exercise on M1 markers (IL-12, IL-23, Nos2) was not clear (Table 2) [15]. Furthermore, administration of the tumorigenic drug dimethylbenz(a)anthracene for six weeks to mice, which was followed by eight weeks of swimming 5 days/week in unknown intensity (Table 1), revealed higher appearance of the M1 macrophage phenotype in the peritoneum and simultaneous decrease in tumor growth at the end of the study (Table 2) [10]. Conversely, the control group had higher presence of the M2 macrophage phenotype in the peritoneum [10]. Overall, although the training parameters among studies were dissimilar, the few data available suggest that exercise reduces the total count of macrophages and enhances the polarization of the M1 instead of the M2 phenotype in mice with cancer, possibly leading to tumor suppression.

### 3.2. The Role of Neutrophils

Neutrophils are known to be important in the modulation of cancer inflammation, displaying both suppressive and progressive behaviors in tumor development. A high density of neutrophils in tumors is associated with both positive and negative prognosis in different cancer types [16]. Therefore, differentiation between the neutrophil phenotypes N1 and N2 may be significant in defining neutrophils’ function in cancer progression [16]. Although neutrophils’ number in the peripheral circulation is increased and remains elevated for a few hours post-exercise [17], their effect on tumor progression remains poorly examined, with only two relevant studies found.

Six weeks of swimming 5 days/week for 1 h/day at 50% of maximal workload (Table 1) in mice with Ehrlich tumors reduced tumor growth and tumor weight, with a concomitant reduction in neutrophil accumulation, although neutrophil polarization in N1 or N2 phenotypes was not assessed (Table 2) [13]. Similarly, two weeks of daily running 3 h/day or until fatigue at gradually increased intensity (20 to 40 m/min at 5% grade, Table 1) in mice with lymphoma impeded tumor growth, although without reduction of tumor volume, and lower tumor infiltration of neutrophils on the 6th and 10th day, compared to control mice [12]. Finally, 10 weeks of daily voluntary wheel running (about 6 km/day, Table 1) in mice injected with I3TC cancer cells on the second week inhibited tumor growth, while intratumoral neutrophil infiltration was not altered (Table 2) [14]. To our knowledge, no other studies have evaluated the effect of exercise on tumor neutrophil infiltration.

### 3.3. The Role of NK Cells

Natural killer cells are lymphocytes of innate immunity with major anti-tumor activity [18]. Dendritic cells [19] and the tumor growth factor platelet-derived growth factor (PDGF)-DD [20] have been proposed to have roles in NK cell-mediated anti-tumor immunity. Physical exercise triggers NK mobilization, with an increased number of NK cells detected in the circulation during exercise [17], which is mostly attributable to the stimulation of catecholamines and of the IL-15, IL-7, and IL-6 myokines [21].

In a mouse model of breast cancer, voluntary (mean distance 6 km/day, Table 1) wheel running, two weeks before and eight weeks after injection of cancer cells, inhibited tumor growth, even though NK tumor infiltration was not changed [14]. In a melanoma mouse model subjected to four weeks of voluntary wheel running (mean distance 4.1 km/day, Table 1) before the injection of tumorigenic (B16) cells, tumor development was decreased, after two more weeks of exercise, by nearly 60% compared to the control group, which was negatively correlated with increased NK tumor infiltration [22]. Also, increased levels of mRNAs related to NK activation (for example, IL-15), was detected in the exercise group, while NK cytotoxicity was not altered in both groups [22]. A negative correlation between increased infiltration of NK and tumor growth was also detected in metastatic B16 lung tumors in the same sample (Table 2) [22]. Interestingly, replication of these procedures in NK-depleted mice showed similar tumor progression between the exercise and the control groups. In another study, 4T1 carcinoma cells were injected in mice and let grow for 22 days, while wheel running was initiated 20 days before injection. Exercising mice performed 60 min/day daily of running at 6 m/min velocity (Table 1) and showed 36% lighter average tumor weight and increased NK tumor infiltration compared to control mice (Table 2) [23]. These data were obtained with different training parameters, however and may suggest that exercise mobilizes NK cells and directs them into the tumor microenvironment, impairing tumor growth.

The effect of exercise on NK cell activation against tumor growth is shown to be superior compared to that of other immune-related pathways. More specifically, voluntary wheel running on an average distance 4.1 km/day in tumor-bearing mice reduced tumor growth by about 60%, while a similar exercise protocol in mice lacking NK cells did not [22]. The exercise-induced mobilization of cytotoxic immune cells depends on the appearance of β2-adrenergic receptors, which are linked to exercise-secreted epinephrine. The higher appearance of β2-adrenergic receptors on NK cells compared to CD8^+^, B, and CD4^+^ cells could explain the increased influence of the exercise—NK cells interaction on tumors suppression [24]. Indeed, 42 days of daily wheel running for 60 min/day at 6 m/min (Table 1) induced enhancement of NK tumor infiltration in mice, which was linked to increased serum epinephrine levels [23]. In addition, adrenergic blockade by propranolol prevented NK infiltration and tumor suppression in exercising (voluntary wheel running, 4.1 km/day on average, Table 1) mice [22]. The clear effect of exercise on NK cells indicates that exercise could be combined with cancer immunotherapies which are based on NK cell functions.

### 3.4. The Role of T Lymphocytes

T and B cells are the main types of lymphocytes in adaptive immunity. The number, cytotoxicity, and tumor infiltration of B cells do not change after six weeks of voluntary wheel running (mean distance 4.1 km/day, Table 1) in tumor-bearing mice [22]; thus, their contribution to tumor suppression in response to any exercise training remains to be elucidated. Naïve T lymphocytes originate from the thymus and mature by responding to signals of innate immune cells, having major importance in anti-cancer immunity [25]. Indeed, tumors of athymic mice (with unfunctional T lymphocytes) were larger compared to tumors of normal mice [22]. The link between acute or chronic exercise, T lymphocytes, and tumor growth/suppression is discussed in the following paragraphs, according to the available data.

#### 3.4.1. Acute Exercise and CD3^+^/CD8^+^/CD4^+^/γδ Lymphocytes

Acute exercise affects homing of lymphocytes, causing a transient redeployment of CD3^+^ among body organs. The most well studied human tissue for exercise-induced changes of lymphocytes is blood. CD8^+^ T lymphocyte counts are temporarily increased by 118% in blood immediately after 20 min of cycling at 85% of maximal workload in healthy humans [26], an observation described as “lymphocytosis” [17,24]. The following (1–3 h later) reduction in their blood count below baseline is called “lymphodepletion” and is possibly the result of a second redistribution [17]. Hours later, the number of lymphocytes in the blood returns to the baseline levels [24]. Lymphocytosis and lymphodepletion also appear in other T lymphocyte subsets such as γδ T [27] and CD4^+^ [24].

Redistribution of immune cells is suggested to enhance immune surveillance, targeting even cancer suppression [4]. Human serum samples of cancer survivors collected immediately after an exercise bout were significantly more efficient in the reduction of cancer cell counts in vitro, compared with serum collected before the exercise bout [28,29]. This reduction in cancer cell counts concurred with lymphocytosis, even though there are no verifiable data to support the mechanistic link between the two processes. Similarly, γδ T cell counts were increased exactly post-exercise and had higher ex vivo expansion and higher ex vivo antitumor activity against U266 cancer cells, in healthy individuals [30]. Interestingly, serum collected from cancer survivors after longitudinal training did not reduce cancer cell number in vitro [28,29], implying that the temporal, not the chronic, increase in T lymphocyte counts may have the anticancer effect. The effect of acute exercise in treating cancer cells is of high importance, suggesting that a longitudinal anti-cancer effect of exercise occurs by the additional effect of each single exercise session, without necessarily inducing long-term adaptations against cancer [28].

A recent study aimed to connect the inhibition of tumor growth after long-term exercise with the acute exercise-induced mobilization of immune cells in mice with breast cancer. The authors reported that an acute treadmill running test with gradually increasing intensity until exhaustion enhanced the activation of CD8^+^ cells (Granzyme B expression), which was triggered by lactate production, and altered CD8^+^ metabolism, elevating anti-cancer efficiency [14]. Nevertheless, the connection between responses of immune cells to acute exercise and tumor status needs further investigation.

#### 3.4.2. Longitudinal Exercise and CD3^+^/CD8^+^ Lymphocytes

Exercise training for about 10 weeks is known to reduce tumor growth in mice, with more favorable effects in mice with higher training status subjected tumor inoculation a few weeks after exercise initiation [31]. However, are T lymphocytes linked to this effect? A high density of “tumor-infiltrating lymphocytes”—T lymphocytes detected into the tumor microenvironment mostly composed of CD8^+^ T cells—is a prognostic biomarker for overall survival in many human cancers, for example, breast cancer [2] and lung cancer [32]. In a rodent cancer model with B16 melanoma, longitudinal wheel running increased tumor infiltration of CD3^+^ [22]. Increased tumor infiltration of CD3^+^ was also found on the 10th day of a two-week treadmill running protocol (Table 1) using the EL-4 tumor mice model [12]. This increase could potentially explain the reduced tumor volume in exercising mice compared to sedentary mice at this exact time point, although this finding was absent before or after the 10th day of training (Table 2) [12]. Twelve weeks of treadmill running (Table 1) using a mice model with colon cancer reduced tumor burden and increased the expression of CD8^+^ in an intestinal tumor model (Table 2) [15]. In mice with breast cancer, voluntary wheel running two weeks prior and eight weeks after tumor injection (Table 1) induced a significant increase in CD8^+^ tumor infiltration and a reduction (>70%) in tumor growth (Table 2) [14]. In addition, athymic [33] or CD8^+^-depleted [14] exercising mice had similar tumor development with respect to normal non-exercising mice, while wheel running prevented tumor development in normal exercising mice [14,33]. Hence, it is suggested that exercise training increases tumor infiltration of CD3^+^/CD8^+^, which may be potentially linked to tumor suppression.

However, tumor reduction in exercising mice was parallel to increased CD8^+^ activation (CD56 expression) and reduction in tumor infiltration of myeloid-derived suppressor cells, but tumor infiltration of CD8^+^ was not altered [34]. In this study, 4T1 carcinoma cell inoculation started 8 days before initiation of treadmill running, which was performed at 18 m/min for 22 days, 30 min/day, 5 days/week (Table 1). Moreover, in one study, 6 weeks of voluntary wheel running for about 4.1 km/day in athymic mice resulted in similar decreases in tumor development as in normal exercising mice [22]. According to these studies, the connection between exercise-induced increase of tumor infiltrating lymphocytes after long-term aerobic exercise and tumor suppression in rodents is not strongly supported. This contradiction may be explained by the nature of the experimental procedures followed. For instance, preparation and mobilization of cytotoxic T cells necessitate more time than those of innate immune cells, implying their later involvement in fighting cancer; therefore, their activity is not evident in rapidly progressive mice tumors. Moreover, in some of these studies, exercise training was initiated two to six weeks before tumor injection (Table 1) [14,22,23,35], suggesting that the plausible mobilization of innate cells created an anti-tumor environment. In one of these studies [22], when exercise training (six weeks, voluntary wheel running, 4.1 km/day, Table 1) started at the same time as tumor incubation, tumor burden was not altered [36]. Also, dissimilarities in mice models, tumor type, and type, duration, and intensity of exercise training may explain the above differences. Nevertheless, the idea for combined exercise and T cell-based immunotherapies in humans [36,37] is also supported by the positive role of exercise in anti-tumor T cell activity [12,14,15], as mentioned in the previous paragraph.

#### 3.4.3. Longitudinal Exercise and CD4^+^ Lymphocytes

The effect of exercise on the T helper (CD4^+^) subtype population (Th1 and Th2) has been assessed after an interval treadmill running protocol in mice with 4T1 carcinoma [35]. The mice in this study started exercise six weeks before cancer induction and continued for six more weeks (Table 1). Spleen INF-γ and IL-4 production were evaluated as markers of Th1 and Th2 activation, respectively [35]. The levels of cytokines were not altered significantly in response to exercise, while tumor development decreased only in exercising mice [35]. Also, CD4^+^ infiltration was not altered in a breast cancer mice model when exercise started two weeks before cancer cells’ injection (Table 2) [14]. These findings indicate that exercise-induced tumor suppression was not affected by changes in the CD4^+^ population.

#### 3.4.4. Longitudinal Exercise and Tregs

Regulatory T cells are involved in an immunosuppressive mechanism with a vital role in healthy populations, preventing autoimmune diseases or allergies. Despite this advantageous function, tumors produce cytokines in order to recruit Tregs, aiming to suppress the anti-tumor immune response [38]. Indeed, high tumor infiltration of FoxP3^+^ Tregs is correlated with poor prognosis in many types of cancer [39]. Also, recent research promotes cancer immunotherapies targeting Treg receptors [40]. 

Eight weeks of wheel running 5 days/week with gradually increasing duration (10 to 26 min/day) and gradually increasing intensity (6 to 33 m/min, Table 1) in mice injected with 4T1 carcinoma cells revealed a higher intratumoral CD8^+^/FoxP3^+^ ratio, longer overall survival, and reduced tumor size compared with control mice. Repetition of the experimental design in athymic mice emphasized the importance of Tregs, as tumor size increased similarly in exercise and control groups [33]. Similarly, in a rodent model with an intestine tumor, 12 weeks of treadmill exercise 6 days/week for 1 h/day at 15 m/min with −5% grade reduced tumor growth and decreased FoxP3 expression in intestine tumor cells (Table 2) [15]. In conclusion, exercise training may have beneficial effect on Tregs modulation in tumors, which may be linked to a reduction in tumor development. Yet, exercise prescriptions in terms of intensity, duration, and frequency, need to be standardized.

## 4. Conclusions

**Figure 1:**
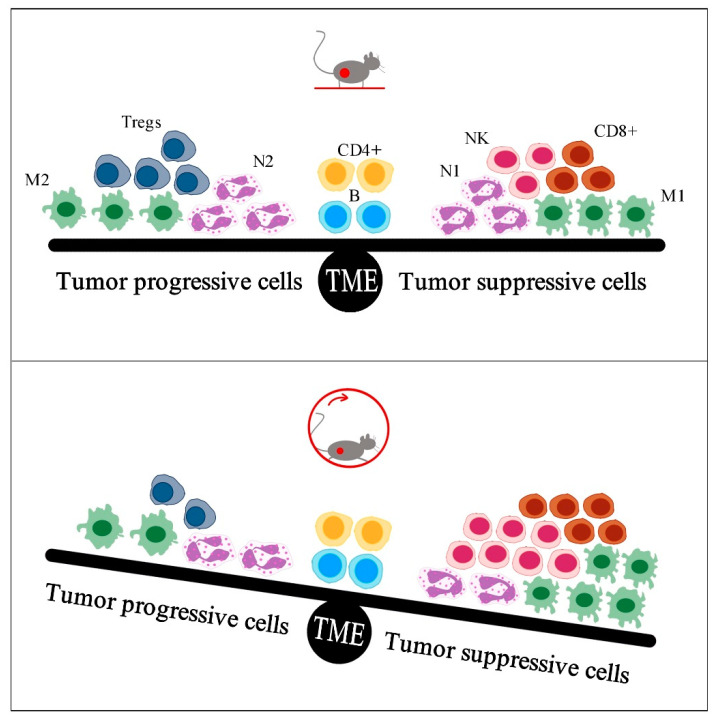
Exercise is shown to enhance infiltration of tumor-suppressive immune cells and to reduce infiltration of tumor-progressive immune cells, preventing tumor growth. TME: tumor microenvironment, M2: macrophages type 2, Tregs: regulatory T lymphocytes, N2: neutrophils type 2, CD4+: T helper lymphocytes, B: B lymphocytes, N1: neutrophils type 1, NK: natural killer cells, CD8+: cytotoxic T lymphocytes, M1: macrophages type 1.

The present review presents a small number of studies which investigated the effect of exercise on tumor growth via changes in tumor immunity, mainly in rodent models. The main conclusion of these studies is that exercise provides beneficial anti-tumor immune responses, such as infiltration of macrophages, neutrophils, NK cells, and T lymphocytes (Table 2). Specifically, exercise reduced the total count of macrophages and induced a favorable polarization to the M1 rather than to the M2 phenotype in tumors of exercising mice, which was accompanied by reduced tumor development [10,12,13,15]. Similarly, exercise resulted in decreased tumor accumulation of neutrophils, which coincided with diminished tumor growth [12,13]. Exercise also resulted in increased tumor density of NK cells, which was negatively associated with tumor burden [22,23]. Also, long-term exercise increased CD8^+^ or CD3^+^ infiltration [12,14,15,22] or activation [34], which contributed to tumor suppression, while it increased the CD8^+^/FoxP3+ ratio in tumors [33] and might diminish FoxP3^+^ tumor infiltration [15], leading to tumor suppression. Overall, exercise enhances the infiltration of tumor-suppressive immune cells and reduces the infiltration of tumor-progressive immune cells, preventing tumor growth (Figure 1). However, the association between exercise anti-tumor effects and changes in tumor immunity has not been explored in all immune cell subtypes, such as neutrophils (N1 and N2), dendritic cells (DC1 and DC2), and the subtypes of T lymphocytes (naïve, senescent, memory, αβ, γδ).

The above changes in tumor infiltration of immune cell subtypes were not noticed simultaneously in all studies (Table 2), possibly because of differences in mice, tumors, or exercise protocols (Table 1). Exercise parameters like intensity frequency, total duration, duration per session, and mode seem to have key roles in the effects of exercise in tumor metabolism of tumor-bearing mice [41]. Indeed, six weeks of swimming 5 days/week for 1 h/day reduced tumor growth when performed at 50% of maximal workload but not when performed at 80% [13]. In another study, tumor-bearing mice performed daily wheel running for 44 days, 60 min/day at 6, 10 or 15 m/min, and it was concluded that the higher the intensity, the higher the decrease of tumor weight, although the reduction in tumor burden was not significant between 10 and 15 m/min [23]. The conflict between the two studies [13,41] emphasizes that other parameters such as mode, duration, and frequency of exercise are also important. Exercise duration per session is also vital [42]. Daily exercise <60 min/day has a significantly higher impact on tumor reduction after exercise training in mice, compared to >60 min/day or voluntary wheel running [31]. Unfortunately, the dissimilarity among the training protocols used in the respective studies renders difficult the direct comparison of the results.

In conclusion, few studies have examined the possible connection between exercise-induced changes in tumor immunity and tumor growth. To date, research data support the idea that physical exercise can be beneficial in anti-tumor immune responses, although more research is needed to evaluate the type, intensity, duration, and frequency of exercise training. Exercise is shown to improve infiltration of tumor-suppressive immune cells and to reduce infiltration of tumor-progressive immune cells, preventing tumor growth (Figure 1). Moreover, there is initial evidence to formulate the hypothesis of an enhancement of immunotherapy outcome when supplemented with systematic physical exercise.

## Figures and Tables

**Table 1:** Training parameters of the included studies.

		Exercise					
No	Study	Type	Duration		Frequency	Per Session	Intensity
			Pre	After			
			Inoculation				
10	Abdalla et al., 2014	swimming		8 weeks	5 days/week	unknown	unknown
12	Zielinski et al., 2004	treadmill running		2 weeks	daily	135 min/day on average	Gradually Increasing 20–40 m/min 5% incline
13	Almeida et al., 2009	swimming		6 weeks	5 days/week	1 h/day	50% of max workload
14	Rundqvist et al., 2020	wheel running	2 weeks	8 weeks	daily	6 km/day on average	voluntarily
15	McClellan et al., 2014	treadmill running		12 weeks	6 days/week	1 h/day	15 m/min −5% incline
22	Pedersen et al., 2016	wheel running	4 weeks	2 weeks	daily	4.1 km/day on average	voluntarily
23	Wang et al., 2020	wheel running	20 days	22 days	daily	1 h/day	15 m/min
33	Hagar et al., 2019	wheel running		8 weeks	5 days/week	10–26 min/day Gradually increasing	Gradually Increasing 4–22 m/min
34	Wennerberg et al., 2020	treadmill running		22 days	5 days/week	30 min/day	18 m/min
35	Shamsi et al., 2019	treadmill running	6 weeks	6 weeks	5 days/week	10 sets 2 min:2 min exercise/recovery	70%:50% of VO_2max_ exercise/recovery

**Table 2:** Exercise-induced changes in tumor infiltration of macrophages (M), neutrophils (N), natural killer cells (NK), total T lymphocytes (CD3^+^), cytotoxic T lymphocytes (CD8^+^), T helper lymphocytes (CD4^+^), and regulatory T lymphocytes (Tregs) and their impact on tumor growth in mice.

No	Study	Tumor	Exercise Duration	Tumor Infiltration							Tumor Growth
				M	N	NK	CD3^+^	CD8^+^	CD4^+^	Tregs	
10	Abdalla et al., 2014	Drug DMBA	8 weeks	↑(M1)							↓
12	Zielinski et al., 2004	EL-4 lymphoid	2 weeks	↓	↓		↑				↓
13	Almeida et al., 2009	Ehrlich tumor	6 weeks	↓	↓						↓
14	Rundqvist et al., 2020	I3TC cells	10 weeks	—	—	—		↑	—		↓
15	McClellan et al., 2014	intestine tumor	12 weeks	↓				↑		↓	↓
22	Pedersen et al., 2016	B16 melanoma	6 weeks			↑	↑				↓
		B16 lung *	6 weeks			↑	—				↓
23	Wang et al., 2020	4T1 breast	44 days			↑					↓
33	Hagar et al., 2019	4T1 breast	8 weeks							↓(CD8/ FoxP3)	↓
34	Wennerberg et al., 2020	4T1 breast	22 days					—(↑CD56)			↓
35	Shamsi et al., 2019	4T1 cells	12 weeks						—		↓

DMBA: dimethylbenz(a)anthracene, M1: macrophages 1, ↑: increase, ↓: reduction, —: no change, * metastatic tumor.

## Data Availability

Data is contained within the article. The data presented in this study are available in Table 1 and Table 2.
